# Experimental Configuration to Determine the Nonlinear Parameter *β* in PMMA and CFRP with the Finite Amplitude Method

**DOI:** 10.3390/s19051156

**Published:** 2019-03-07

**Authors:** Antonio Callejas, Guillermo Rus

**Affiliations:** 1 Department of Structural Mechanics, University of Granada, 18071 Granada, Spain; grus@ugr.es; 2 Instituto de Investigación Biosanitaria, ibs.GRANADA, 18012 Granada, Spain; 3 Excellence Research Unit, “Modelling Nature” (MNat), University of Granada, 18071 Granada, Spain

**Keywords:** nonlinear parameter *β*, finite amplitude method, harmonic generation, polymethylmethacrylate, carbon fiber reinforced polymer

## Abstract

Parameters to measure nonlinearity in polymethylmethacrylate (PMMA) and carbon fiber reinforced polymer (CFRP) materials have been determined with nonlinear ultrasound (NLUS). The nonlinear parameter β has been determined using the variation of the Finite Amplitude Method (FAM) with harmonic generation. Using this as a reference, the first contribution of this work consists of deducting the experimental configuration necessary to measure this nonlinear parameter in a correct and feasible way. Excitation level, frequency of the wave generated, number of cycles analysed and the distances transducer-specimen and specimen-hydrophone have been determined in both materials. The second contribution is a semi-analytical model that allows to obtain the nonlinear parameter in materials by removing water contribution and considering geometric and viscous attenuation, using the data obtained in an immersion tank. Finally, an application of this model has been carried out in PMMA in order to determinate the nonlinear parameter in this material. From the results, we confirm that the configuration determined in this paper to obtain the parameter β decreases the noise in the measurements.

## 1. Introduction

Within the field of ultrasonic nonlinearity, different experimental techniques have been developed to measure the nonlinearity: Finite Amplitude Method (FAM) based on harmonic generation, Nonlinear Elastic Wave Spectroscopy (NEWS), including Nonlinear Resonant Ultrasound Spectroscopy (NRUS), and Nonlinear Wave Modulation Spectroscopy (NWMS) and Dynamic Acousto-Elasticity (DAE) [1].

Measuring the amplitudes of harmonics is commonly referred to as the finite-amplitude (FA) method, initially developed by Breazeale and Thompson (1963) [2]. The nonlinear coefficients are usually determined by measuring the second-harmonic generation and sometimes higher harmonics, and can be used to characterize acoustic nonlinear properties of gases, liquids, and solids. For this technique, the through-transmission mode in immersion is usually preferred. Instead of using two transducers, it is opportune to replace the receiver by a needle hydrophone (with a nearly linear frequency response), in order to conveniently measure the second and higher-harmonics.

Different wave types have been investigated with this technique, such as shear waves, surface acoustic waves (SAW), lamb waves [3], but above all longitudinal waves. Initially, bulk exploration using nonlinear longitudinal waves has attracted the attention of many authors.

Taking into account that the amplitude of the second harmonic is fundamental to detect early damage, in the case of shear waves propagating in pure isotropic materials it is complicated as the amplitude of the second harmonic is negligible [4]. Bulk waves experiences need double-sided access for the characterization of the specimen, while SAW and lamb ones just need a single-side access. Notwithstanding, the pulse-echo technique using longitudinal waves has slightly been assessed in fluids [5], and metals [6,7]. In such a case, the rebound on a pressure release boundary in most NDT applications and the double interaction make the signal interpretation particularly difficult [8].

The finite-amplitude technique has been shown to be useful for defect detection in ceramics [9], concrete structures [4,10], composites [11], fatigue cracks in metals, such as steels, titanium, and aluminum alloys [12,13]. Such defects are originated in internal stresses, micro-cracks, zero-volume disbonds, and usually precede the main cracking mechanisms and the failure of the material. Therefore, a considerable number of authors have been involved in laboratory experiments to show that cracks and imperfect interfaces can behave in a nonlinear fashion [14,15], and have thus opened new opportunities to detect partially closed cracks that may not be identified by conventional linear methods.

The finite-amplitude method is a relatively straightforward technique to measure the second- and higher harmonic peaks, and thus obtain nonlinear elastic coefficients of a material. The low complexity of the experimental installation could make of this method a low-cost and valuable technology for in-situ industrial applications compared to NRUS, NWMS and DAE techniques. Nonetheless, a practical extraction of the harmonics requires numerous efforts in minimizing the nonlinear distortions from electronic devices and in optimizing the reproducibility of the experiment. Indeed, several factors such as the size of the gap between the specimen and the receiver or the geometrical dispersion of the transducers (inherently related to the focal distance) may have a drastic influence on the measured nonlinear elastic coefficients, and should be analyzed carefully.

Nonlinearity in fluids has been investigated for a long time. The need to investigate this aspect is that several disciplines are interesting in this aspect, disciplines as medicine, engineering, etc. The first fluid that was investigated was air [16]. After that, some investigators studied nonlinearity in other fluids.

Several parameters, in order to measure the nonlinearity in fluids and solids, have been developed, some of them are: β, determined from the ratio of amplitudes of the fundamental and that of the second harmonic generated in the medium under study, B/A, whose relationship with β is: $\beta =1+\frac{B}{2A}$ (A and B are parameters of the adiabatic expansion of pressure and the coefficient of the quadratic, respectively), Γ, etc. [17]. These parameters are based on the same principle, the relationship between the fundamental harmonic and the second harmonic, concretely between the fundamental and the square of the second harmonic (β).

Some researches have determinated the nonlinear parameter B/A in water, liquid mixtures, water with high pressure and temperature, biological media, etc. [18,19,20,21,22,23,24,25]. Water is the fluid that has been investigated more and it is the medium by which the wave propagates in an immersion tank. The propagation of a finite-amplitude plane wave through an acoustic nonlinear medium introduces distortions, resulting in the generation of higher harmonics. The acoustic nonlinearity observed as appearance of harmonics in ultrasound propagation is a consequence of the deviation from perfect linear elasticity of the compressional mechanical constitutive law. The propagation of the wave through this medium causes the generation of higher harmonics. Experimentally, the acoustic nonlinear parameter of water was determinated, obtaining the following results: β=3.5±0.1 [21], B/A=6.2±0.6 [18] and B/A=5.2 [20]. These values were determinated at atmospheric conditions. The influence of pressure and temperature on the value was studied by Plantier et al. [22]. The higher pressure values, the higher nonlinear parameter and vice versa. Zhang et al. [26] proposed a novel method to determine the nonlinearity in fluids and solids using attenuation and diffraction corrections. For water measurements, this procedure yields the acoustic nonlinear parameter close to previous studies (β=3.5). One year later, Jeong et al. [27] presented a novel approach to determine the nonlinearity parameter using an optimized data fitting method, based on the quasilinear theory of the Khokhlov–Zabolotskaya–Kuznetsov (KZK) equation. Li et al. [28] presented analytical and experimental techniques for absolute determination of the acoustic nonlinearity parameter (β) in fluids using focused transducers. More recently, Jeong et al. [29] described the acoustic nonlinearity parameter (β) determination for fluids using a pulse-echo method with the stress-free boundary.

Using the Finite Amplitude Method (FAM), there are evidences about nonlinear parameter in fluids and PMMA, however there are little evidence in CFRP. Respect to polymethylmethacrylate material (PMMA), there are few references about the nonlinear parameter β using the Finite Amplitude Method. Renaud et al. [30] found β=14 for Plexiglas (PMMA), whereas the usual value is 7.5 [30]. This overestimation was probably due to reflections inside the Plexiglas sample. β values for the CFRP are hardly comparable, since this material may strongly depend upon the manufacture process, on the properties of each component, and on the laminate stacking sequence.

There is a gap within the experimental configuration in the data obtained from p-waves in an immersion tank [30]. This field has not been considered by the researchers yet and it is a very important subject. As an example, Idjimarene et al. [31] state that the nonlinear indicator is dependent on the position of the receiver, and it is sensitive to the level of noise. The main contribution of this paper states a semi-analytical approach to eliminate the dependence of experimental settings on β measurements.

Regarding the structure of this work, the nonlinear parameter β has been obtained using a variation of the FAM with harmonic generation. In order to measure the nonlinear parameter in a correct and feasible way in an immersion tank, it has been deducted the experimental configuration for both materials, PMMA and CFRP (excitation level, frequency of the wave generated, number of cycles analysed and the distances transducer-specimen and specimen-hydrophone). Then, a semi-analytical model that allows to obtain the nonlinear parameter in materials by removing water contribution and considering geometric and viscous attenuation was presented. Finally, the nonlinear parameter β has been obtained in PMMA by using the presented semi-analytical model.

## 2. Methodology

In this section, the experimental setup (Section 2.1) used to measure in an immersion tank is shown. After that, it is presented the devices description in Section 2.2. The specimens used in this paper have the characteristics indicated in Section 2.3. In Section 2.4 the variables, in the context of the test, are described. Finally, the equation to determinate the nonlinear parameter β and a semi-analytical approach are presented in Section 2.5 and Section 2.6 respectively.

### 2.1. Experimental Setup

The specimens were excited with a range of frequencies around the center frequency of the transducer, to see how the nonlinearity varied with the range of frequencies. For each frequency, the wave generator (Agilent 33250 A) sent various excitation energies that were amplified (AR 150 A) before reaching the transducer (Olympus Panametrics—NTD 5 MHz).

After that, the wave generated by the unfocused transducer (which transforms the electrical signal into acoustic signal), travels in the immersion tank through the water to the specimen (PMMA or CFRP). This signal, which is attenuated by water and transmission coefficient water-specimen, interacts with the nonlinearity of the material, generating a wave rich in harmonics. After crossing the material, the wave travels back through the water layer to reach the hydrophone (ONDA HNR-0500), which converts acoustic signal (wave) into an electrical signal, pre-amplified (Olympus 5676) and displayed on the oscilloscope (HDO 4034).

**Figure 1:**
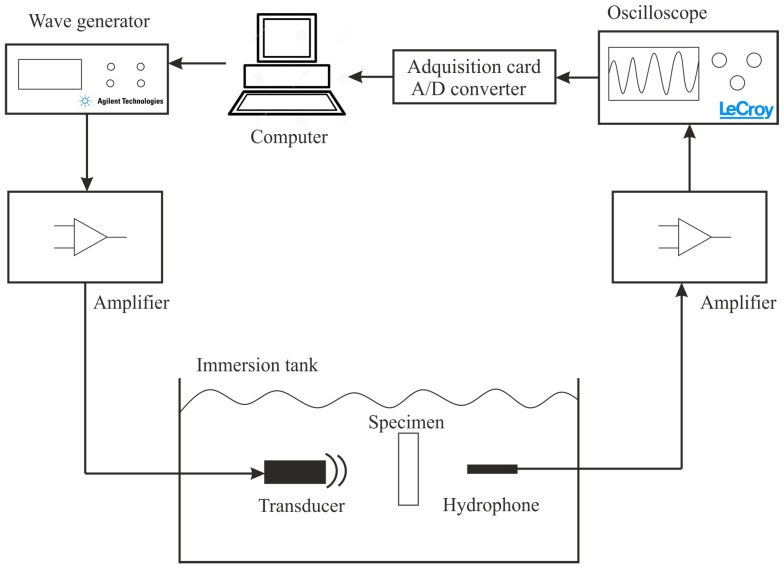
Experimental setup.

The specimen-hydrophone distance varies while keeping fixed the distance between the transducer and the specimen. Finally, the recorded signal is processed to obtain an apparent nonlinearity parameter which takes into account the electrical nonlinearity, water nonlinearity and specimen nonlinearity (Figure 1).

The used method is called Harmonics Generation Method, which consists on analyzing the first and second harmonics generated by the materials in which the wave is propagated.

### 2.2. Materials Description

The completion of the trials was carried out with two specimens, polymethylmethacrylate (PMMA) and carbon fibre reinforced polymer (CFRP). The first one has been chosen as a model material to validate the semi-analytical approach. The CFRP specimen was chosen as a complex material in terms of internal structure.

**Figure 2:**
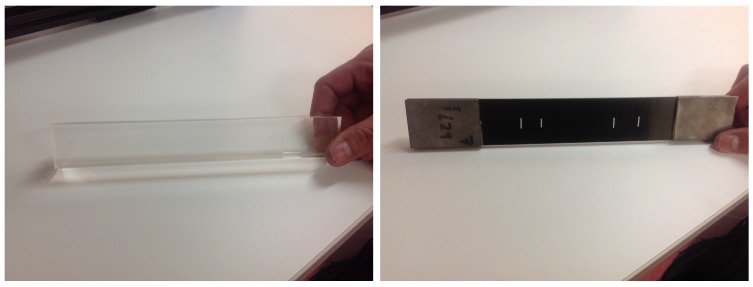
Polymethylmethacrylate material (PMMA) and carbon fibre reinforced polymer (CFRP) specimens.

The first, PMMA (Figure 2, left), whose molecular formula is $C_{5}O_{2}H_{8}$, is the most transparent plastic, with a transparency of about 93%, a thickness of 20 mm and a density of 1190 kg/m${}^{3}$. Within two materials mentioned, this is a very homogeneous material against carbon fibre.

The second material is CFRP (Figure 2, right), with a stacking sequence which corresponds to a ${\left[0/90\right]}_{4s}$ lay-up. During lay-up, the laminates were compacted every four layers in the stacking sequence by applying vacuum for 15 min. Curing was realized in an autoclave at 177 ${}^{\circ }$C for three hours with a pressure of 7 bar. It has a density of 1800 kg/m${}^{3}$ and a thickness of 2 mm. This previous CFRF plate was previously subjected to stress—fatigue load in tension-compression (400,000 cycles) with the objective of testing a material simulating the real conditions during its useful life. Fatigue testing was conducted with a servo-hydraulic Instron/Schenk 100 kN fatigue testing machine with hydraulic clamps at a stress ratio of R = −1. The clamping pressure was set according to loading forces.

### 2.3. Variables

In order to obtain the correct experimental configuration to measure the nonlinear parameter β in an immersion tank, the variables under study are:

- **Excitation level**. This is the excitation energy sent from the transducer to the hydrophone, going through the specimen (PMMA or CFRP). The signal voltage is produced in the wave generator (Agilent 33250 A) and amplified 27.5 dB by the amplifier (Amplifier Research 150A 100B). Three excitation levels were considered: 320 mV, 240 mV and 160 mV. This choice is based on the previous experience for generating nonlinearity.
- **Frequency**. It is generated in the transmitter transducer. It depends on the type of transducer. The transducer used has a central frequency of around 5 MHz, so it was decided to do a frequency sweep around this central frequency. This sweep was done as follows: from 4 MHz to 7 MHz increasing that frequency by 0.1 MHz. It was expected to get a accurate information about the correct frequency.
- **Distance**. The distance was varied between specimen-hydrophone, while maintaining a fixed distance between the transducer-specimen. This last distance is established because of the effects of the near field. The distance specimen-hydrophone is varied from 0.5 mm to 50.5 mm. The step is defined in 1 mm. This movement can be automatized because of the mechanical arm of the immersion tank controlled in MATLAB with the correspondent libraries for controlling the step-by-step motors.
- **Specimen thickness**. This is important data since the specimen thickness is inversely proportional to the nonlinear parameter β. This was measured with a gauge to ensure this variable with more accuracy.
- **Sampling**. In the sampling, the acquisition card was adjusted with a number of points for each cycles by which the sampling frequency was integer. This was necessary because if this was not done, the FFT in MATLAB was not well done, and may have problems like aliasing and leakage. With this adjustment these problems were avoided.
- **Window variation**. This variable is the time window in which the ultrasonic wave arrives to the hydrophone until a certain number of cycles. Different windows for each frecuency were got. This was done because it was necessary to adjust the number of points analysed by the acquisition card. It was taken the number of points divided by 10 (the number of points that represent the wave) and it was got the number of cycles that the windows are able to capture. The wave region varies in the distance, so the variance was established with an estimation of the retardment with the wave arriving at the hydrophone.
- **Wave region**. The first part of the wave was analysed in that window, trying to avoid undesired interferences which distort the value of the non-linear parameter β. This region depends on the material, for PMMA it was analysed until 150 cycles, nevertheless in the CFRP laminates it was analysed the firsts 30 cycles.
- **Hydrophone sensitivity**. In order to obtain a value of efficient beta (with water and material), it is necessary to obtain the value of the fundamental and second harmonic and each one has a different value of frequency (double), this was corrected with the sensitivity curve of the hydrophone, and each harmonic was treated with different value of this sensitivity.
- **Alignment**. It is important the correct alignment between transducer and hydrophone because a little misalignment causes variations in the nonlinear parameter β.

### 2.4. Theoretical Foundations to Determine the Real Parameter β Considering Geometric and Viscous Attenuation

The contribution that this section shows is the determination of a relationship between the nonlinear parameter β and the amplitude of the fundamental and second harmonic in two points separated by a distance x, considering geometric and viscous attenuation.

In order to solve the wave propagated by a transducer along the center of the path, the $x_{1}$ axis is chosen as aligned with the propagation path, and the transversal components of the displacements are neglected. Thus, the 3D problem of finding $(u_{1}\left(x_{1},x_{2},x_{3},t\right)$, $u_{2}\left(x_{1},x_{2},x_{3},t\right),u_{3}\left(x_{1},x_{2},x_{3},t\right))$ is reduced to a 1D problem of finding the displacement field $\left(u_{1}\left(x_{1},t\right),0,0\right)$ whose solution will be found analytically.

**Figure 3:**
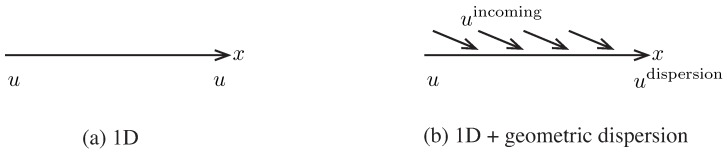
Scheme of inclusion of out of beam components as geometrical dispersion.

A strict application of the former simplification leaves out the effect of the geometric dispersion on the propagation along the axis. This will be shown to be responsible for large deviations that drastically reduce the validity of the formula that relates β with the amplitude of the harmonics. To overcome this, a procedure to include the effect of the geometric dispersion into the 1D formulation is presented. To this end, the compatibility equation is modified to include the incoming or outgoing components from outside the center of the beam, as depicted in Figure 3.

The incoming displacement $u^{\mathrm{incoming}}$ adds continuously a component to the original displacement, both in the $x_{1}$ direction. This can be expressed in the following differential form, where the addition happens gradually by a proportionality factor $2\alpha_{g}$,

(1) $$\begin{array}{c} \frac{du_{1}^{\mathrm{dispersion}}\left(x_{1},t\right)}{dx_{1}}=\frac{du_{1}\left(x_{1},t\right)}{dx_{1}}+2\alpha_{g}\left(x_{1}\right)u_{1}\left(x_{1},t\right) \end{array}$$

This can also be interpreted as a rate of change of the displacement $u^{\mathrm{incoming}}-u$ with respect to the case without incoming wave, proportional to the amplitude *u* as,

(2) $$\begin{array}{c} \frac{d\left(u_{1}^{\mathrm{dispersion}}-u_{1}\right)}{dx_{1}}=2\alpha_{g}u_{1} \end{array}$$

The modified compatibility equation follows straightforwardly, including a new geometric dispersion term,

(3) $$\begin{array}{c} \varepsilon_{11}=u_{1,1}+2\alpha_{g}u_{1} \end{array}$$

The compatibility, constitutive and equilibrium equations become, after assuming directional propagation $u_{2}=0=u_{3}$,

(4) $$\begin{array}{ccc} \mathrm{Compatibility}:\; & \varepsilon_{11}=u_{1,1}+2\alpha_{g}u_{1}=\varepsilon =-3v,\; & \varepsilon_{ij}=0\;\forall \left(i,j\right)\neq \left(1,1\right) \end{array}$$

(5) $$\begin{array}{ccc} \mathrm{Constitutive}:\; & \sigma_{kk}=-p=K\varepsilon +\frac{1}{2}\beta K\varepsilon^{2}+\eta^{v}\dot{\varepsilon },\; & \sigma_{ij}=0\;\forall i\neq j \end{array}$$

(6) $$\begin{array}{cc} \mathrm{Equilibrium}:\; & \rho {\ddot{u}}_{1}=\sigma_{11,1} \end{array}$$

The last four equations can be combined by substitution into a 1D nonlinear wave equation that includes geometrical dispersion correction,

(7) $$\begin{array}{c} \rho {\ddot{u}}_{1}=Ku_{1,11}+2\alpha_{g}Ku_{1,1}+\frac{1}{2}\beta K{\left(u_{1,1}^{2}\right)}_{,1}+\eta^{v}{\dot{u}}_{1,11}+O\left(\beta \delta \right)+O\left(\eta \delta \right) \end{array}$$

where higher order terms O(βδ),O(ηδ) are negligible, and the four relevant terms at the right hand side are the linear compressibility, the geometrical dispersion, the nonlinear compressibility and the viscosity. For the sake of compactness, the direction index 1 will be dropped in the sequel, and the spatial derivative with respect to $x_{1}$ will be denoted by a tilde (i.e., $u_{1,1}=u^{\prime }$).

#### Solution

The solution of Equation (7) is sought as the sum of two attenuating traveling waves at velocity *c* with frequency ω and 2ω respectively, that stand for the fundamental due to linear propagation ($u_{0}$), and the harmonic generated by the nonlinearity ($u_{1}$, which will be shown to be proportional to the degree of nonlinearity β). The complex exponential notation is adopted, where the phase component is omitted without loss of generality,

(8) $$\begin{array}{c} u=u_{0}+u_{1}\;\left\{\begin{array}{c} u_{0}\left(x,t\right)=a\left(x\right)e^{i(kx-\omega t)}\\ u_{1}\left(x,t\right)=b\left(x\right)e^{2i(kx-\omega t)} \end{array}\right. \end{array}$$

Substituting the decomposition above into Equation (7) and neglecting terms of order $O(\beta^{2})$ yields,

(9) $$\begin{array}{c} \frac{\rho }{K}\left({\ddot{u}}_{0}+{\ddot{u}}_{1}\right)=u_{0}^{\prime \prime }+u_{1}^{\prime \prime }+2\alpha_{g}u_{0}^{\prime }+2\alpha_{g}u_{1}^{\prime }+\frac{1}{2}\beta {\left(u_{0}^{\prime 2}\right)}^{\prime }+\frac{\eta^{v}}{K}{\dot{u}}_{0}^{\prime \prime }+\frac{\eta^{v}}{K}{\dot{u}}_{1}^{\prime \prime } \end{array}$$

Recalling that the successive derivatives of the displacement components are,

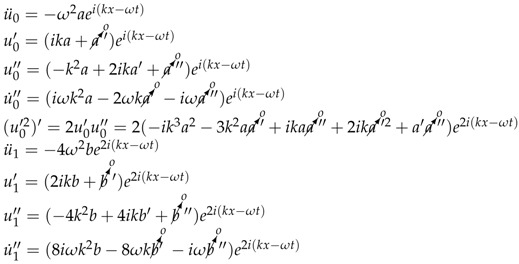

where some terms have been neglected since, for ultrasonic waves the wavenumber is much larger than the viscous or geometric dispersions, $k>>\alpha_{g},\alpha$ (e.g., usual values for the frequency range used in this work are: $k=2\cdot 10^{4}\cdot m^{-1}$, $\alpha =2\times \frac{dB}{m}$). The meaning of α and its relationship with $a^{\prime }$ and $a^{\prime \prime }$ will be understood in short.

Equation (9) should be fulfilled independently for terms propagating as $e^{i(kx-\omega t)}$ as for terms as $e^{2i(kx-\omega t)}$. This implies that the equation can be split into two equalities, of which the first one is,

(10) $$\begin{array}{c} \frac{\rho }{K}{\ddot{u}}_{0}=u_{0}^{\prime \prime }+2\alpha_{g}u_{0}^{\prime }+\frac{\eta^{v}}{K}{\dot{u}}_{0}^{\prime \prime } \end{array}$$

Given a fundamental excitation frequency ω, Equation (10) is satisfied if $\frac{\rho }{K}=\frac{k^{2}}{\omega^{2}}=c^{-2}$, which defines the compressional wave velocity *c* and the wavenumber *k*. Equation (10) transforms into,

(11) $$\begin{array}{c} 0=2ika^{\prime }+2\alpha_{g}ika+\frac{\eta^{v}}{K}ik^{2}\omega a\;\Rightarrow \;a^{\prime }=-\left(\alpha_{g}+\frac{\omega^{2}\eta^{v}}{2\rho c^{3}}\right)a \end{array}$$

which is a differential equation of first order, whose solution is, recalling that $K=\rho c^{2}$, and calling $\alpha =\frac{\omega^{2}\eta^{v}}{2\rho c^{3}}$,

(12) $$\begin{array}{c} a\left(x\right)=a\left(0\right)e^{-(\alpha_{g}+\alpha )x} \end{array}$$

The second equality, which groups terms propagating as $e^{2i(kx-\omega t)}$, is,

(13) $$\begin{array}{c} \frac{\rho }{K}{\ddot{u}}_{1}=u_{1}^{\prime \prime }+2\alpha_{g}u_{1}^{\prime }+\frac{\eta^{v}}{K}{\dot{u}}_{1}^{\prime \prime }+\frac{1}{2}\beta {\left(u_{0}^{\prime 2}\right)}^{\prime } \end{array}$$

which, by removing common factors transforms into,

(14) $$\begin{array}{c} b^{\prime }+\left(\alpha_{g}+4\alpha \right)b=\frac{\beta k^{2}a{\left(x\right)}^{2}}{4} \end{array}$$

which is a differential equation of first order, of the form $y^{\prime }+fy=g$, whose solution is somewhat more complex of the form $y=e^{-\int fdx}\left(\int ge^{\int fdx}dx+c\right)$.

(15) $$\begin{array}{c} b\left(x\right)=b\left(0\right)e^{-(4\alpha +\alpha_{g})x}+\frac{\beta k^{2}a{\left(0\right)}^{2}}{4(\alpha_{g}-2\alpha )}e^{-(2\alpha +2\alpha_{g})x} \end{array}$$

The nonlinear parameter β, considering geometric and viscous attenuation is:

(16) $$\begin{array}{c} \beta =\frac{\left(b\left(x\right)-b\left(0\right)e^{-(4\alpha +\alpha_{g})x}\right)\left(4\left(\alpha_{g}-2\alpha \right)\right)}{k^{2}a{\left(0\right)}^{2}e^{-(2\alpha +2\alpha_{g})x}} \end{array}$$

However, if Equation (13) is approximated by neglecting both viscous and geometric dispersion terms, the following solution is recovered,

(17) $$\begin{array}{c} b\left(x\right)=b\left(0\right)+\frac{\beta k^{2}a{\left(0\right)}^{2}x}{4} \end{array}$$

If the initial amplitude of the second harmonic is assumed to be zero, the standard nonlinearity estimator from the literature is recovered,

(18) $$\begin{array}{c} \beta =\frac{4b(x)}{k^{2}a{\left(x\right)}^{2}x} \end{array}$$

### 2.5. Semi-Analytical Approach

**Figure 4:**
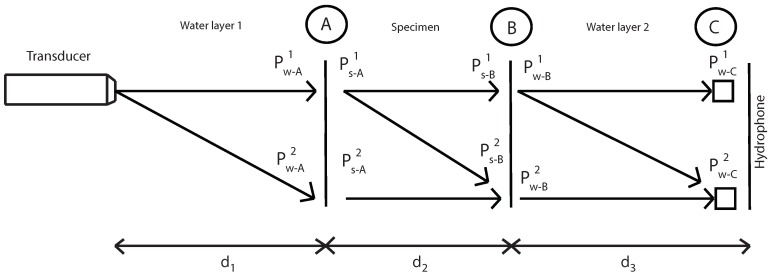
Semi-analytical approach used to extract the nonlinear material’s properties from the measurements.

After determining the correct parametric configuration in an immersion tank, measurements of the nonlinear parameter β have been carried out. The scheme used for determining the nonlinear parameter β is shown in Figure 4.

P is the pressure of the fundamental (superscript “1”) and second harmonic (superscript “2”), “w” subscript indicates water, “s” indicates specimen and $d_{1}$, $d_{2}$ and $d_{3}$ are distances.

The main aim is to determinate the fundamental and second harmonic pressure in A and B inside the specimen. With this values, the nonlinear parameter β will be determinated. Before that, several steps are necessary.

**Figure 5:**
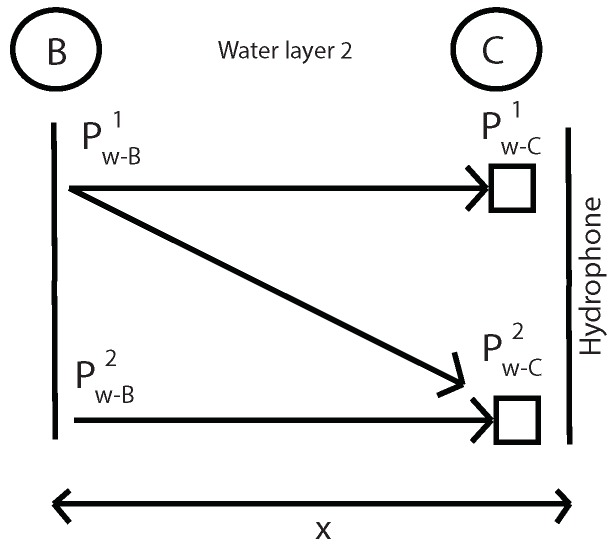
Determination of the β parameter and geometric attenuation in water layer 2 without specimen.

The first step is to measure the fundamental and second harmonics pressure in A, B and C without specimen. With this values in B and C and using Equations (12) and (15) it will be determinated the nonlinear parameter β in water and geometric attenuation in this water layer (Figure 5). Where “k” is the wave number, “α” is the viscous attenuation of water at the fundamental frequency ($\alpha =20\times 10^{-15}\cdot f^{2}$) [32] and “x” is the thickness of water layer 2.

**Figure 6:**
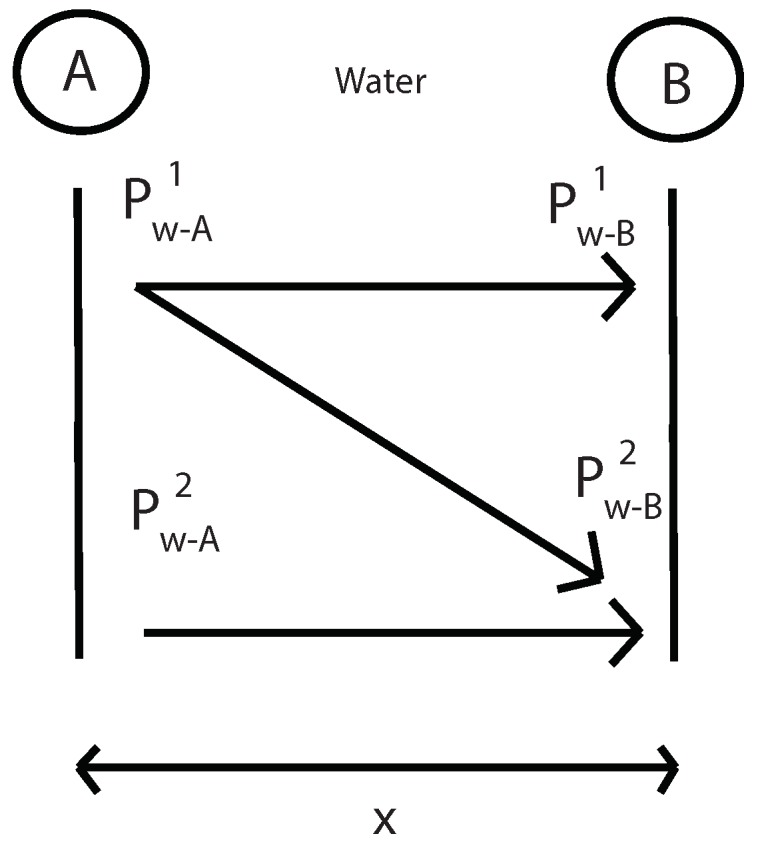
Determination of the geometric attenuation between A and B points without specimen.

Then, using the same Equations (12) and (15), geometric attenuation between A and B point is obtained without the presence of the specimen (Figure 6). Now, “x” is the distance between A and B.

**Figure 7:**
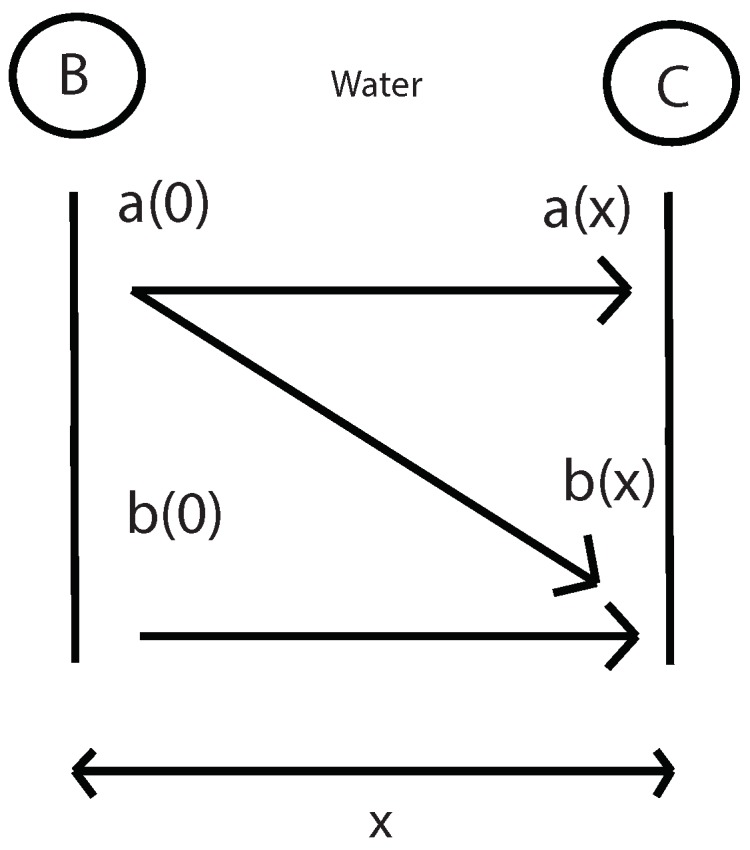
Determination of the fundamental and second harmonic in B by propagating this values from C to B.

The next step is to measure the fundamental and second harmonic pressure in C with the presence of the specimen. With this values and the values of nonlinear parameter β in water and geometric attenuation between B and C (previously calculated), it will be determinated the amplitude of the fundamental and second harmonic in water and in B point (Figure 7), using the equations below:

(19) $$\begin{array}{c} a\left(0\right)=\frac{a(x)}{e^{-(\alpha_{g}+\alpha )x}} \end{array}$$

(20) $$\begin{array}{c} b\left(0\right)=\frac{b\left(x\right)-\frac{\beta k^{2}a{\left(0\right)}^{2}}{4(\alpha_{g}-2\alpha )}e^{-(2\alpha +2\alpha_{g})x}}{e^{-(4\alpha +\alpha_{g})x}} \end{array}$$

The last step, before reaching the goal, is to obtain the values of the fundamental and second harmonic in A and B in specimen by multiplying this values in water for transmission coefficient water-specimen.

The transmission coefficient water-specimen is:

(21) $$\begin{array}{c} T_{w-s}=\frac{2Z_{w}}{Z_{w}+Z_{s}} \end{array}$$

where $Z_{w}$ and $Z_{s}$ are the water impedance and the specimen impedance respectively.

Finally, with the values of fundamental and second harmonic in A and B in specimen and taking specimen attenuation from literature and geometric attenuation calculated before between A and B points, the nonlinear parameter β in specimen, considering specimen and geometric attenuation, is calculated as follows:

(22) $$\begin{array}{c} \beta =\frac{\left(b\left(x\right)-b\left(0\right)e^{-(4\alpha_{s}+\alpha_{g})x}\right)\left(4\left(\alpha_{g}-2\alpha_{s}\right)\right)}{k^{2}a{\left(0\right)}^{2}e^{-(2\alpha_{s}+2\alpha_{g})x}} \end{array}$$

where “x” is the specimen’s thickness.

### 2.6. Specimen Attenuation

In order to compute the nonlinear parameter β in the PMMA specimen, an experimental test has been performed to obtain the specimen attenuation ($\alpha_{s}$). Assuming that the attenuation follows an exponential law as shown in the following equation:

(23) $$\begin{array}{c} \frac{V_{0}}{V_{0}^{{}^{\prime }}}=e^{\alpha_{s}\left(x-x_{0}^{{}^{\prime }}\right)} \end{array}$$

where $V_{0}$ and $V_{0}^{{}^{\prime }}$ are the signal amplitudes at different sample distances and *x* and $x_{0}^{{}^{\prime }}$ are the different distances.

**Figure 8:**
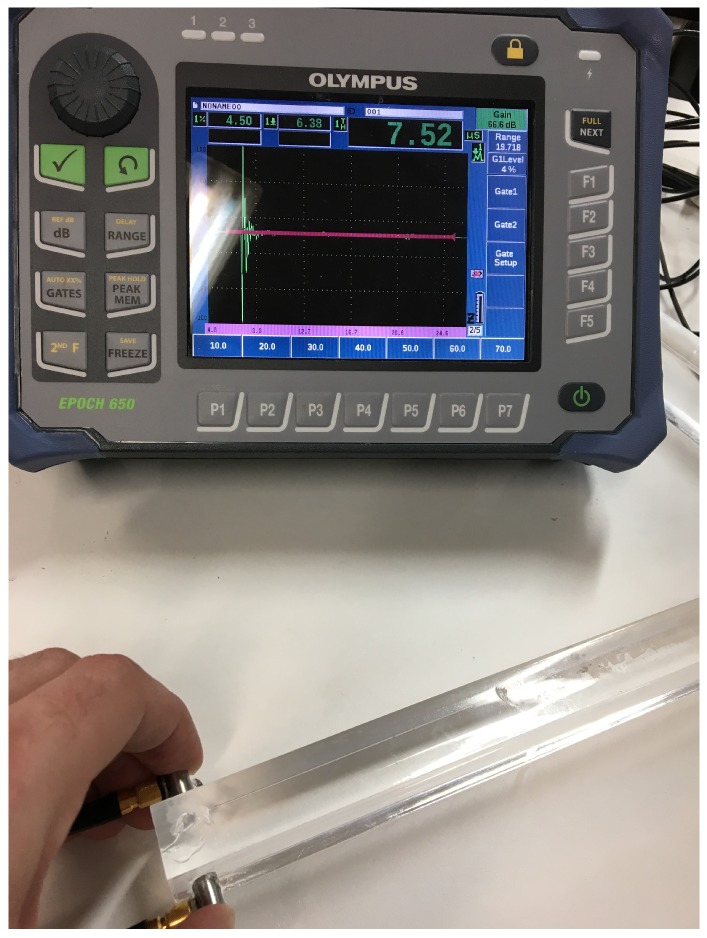
Experimental setup to determine the PMMA attenuation.

The experimental setup is shown in Figure 8. Two 5 MHz transducers (Olympus Transducer V1091, Tokyo, Japan) have been used in order to measure the attenuated signal in direct transmission. The signal is emmitted and received by a pulser-digitizer (Olympus Epoch 650, Tokyo, Japan).

## 3. Results

This section presents the parametric configuration obtained with two specimens: PMMA and CFRP (Section 3.1 and Section 3.2). The final subsection of this section, Section 3.3, presents an application of the semi-analytical approach (Section 2, Section 2.1, Section 2.2, Section 2.3, Section 2.4, Section 2.5, Section 2.6) with PMMA specimen. In the figures shown in this section, “Beta approx” refers to the non-linear parameter of the material without neglecting the non-linearity of the measurement configuration.

### 3.1. PMMA Results

#### 3.1.1. Excitation Level

The excitation level was chosen in a first approximation with three different frequencies (5 MHz, 5.5 MHz and 6 MHz) around the center frequency of the transducer and three different energies (320 mV, 240 mV and 160 mV). A scan was performed in the wave emission direction, between 0.5 mm and 50.5 mm from the specimen.

**Figure 9:**
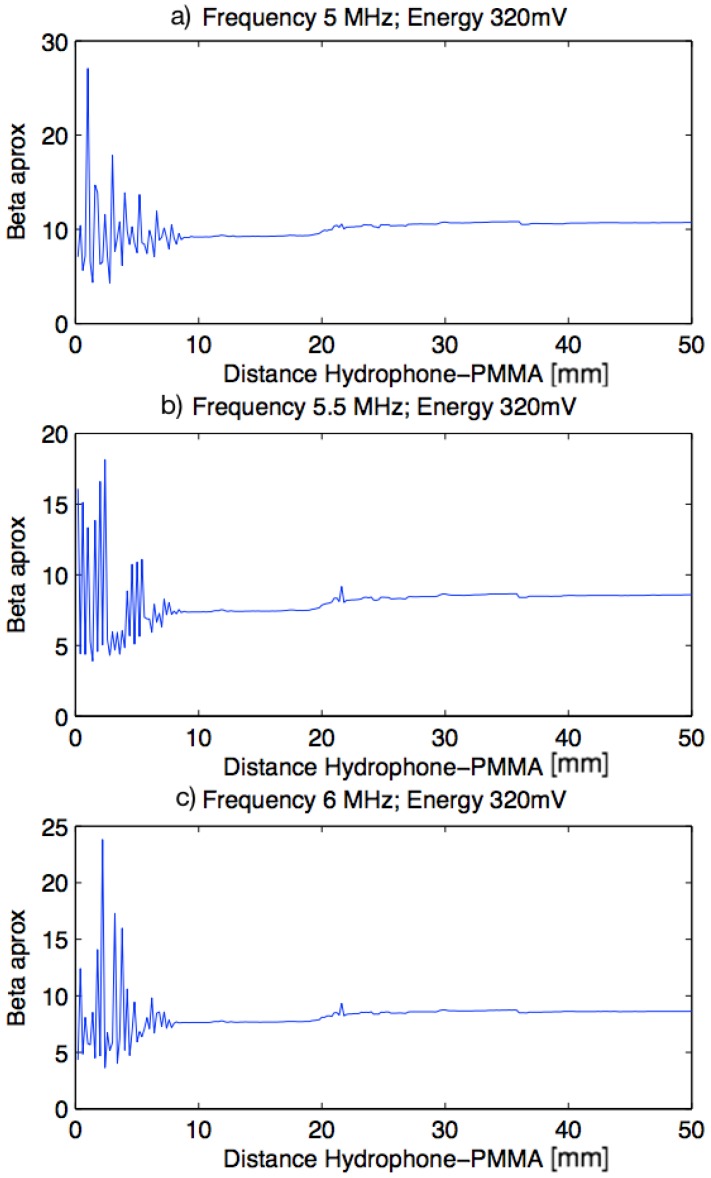
Plots beta versus distance specimen-hydrophone. (**a**) Frequency of 5 MHz and Energy of 320 mV; (**b**) Frequency of 5.5 MHz and Energy of 320 mV; (**c**) Frequency of 6 MHz and Energy of 320 mV.

In the Figure 9 it can be observed that the nonlinear parameter β in the first centimeter has a very high variation. From there, variation is established around a value of beta and this stays until the end of the scanning. Results show the same pattern with a fixed frequency and for the different values of excitation level. By this reason, the excitation level of 320 mV was chosen because the higher excitation level, the higher nonlinearity level obtained, which implies that high order harmonics can be obtained with a higher level of energy.

#### 3.1.2. Frequency

After choosing the excitation level, data from the tests with box-plots were studied. This sort of plot performs a data processing showing the mean and different percentiles of a group of data. In this case, it was analysed the value of the nonlinear parameter β in different intervals of distance specimen-hydrophone: between 0–10 mm, 10–20 mm and 20–30 mm, for 50 cycles analysed and for different frequencies (4–7 MHz) to get information about which frequency has less variance around the central frequency of the transducer.

**Figure 10:**
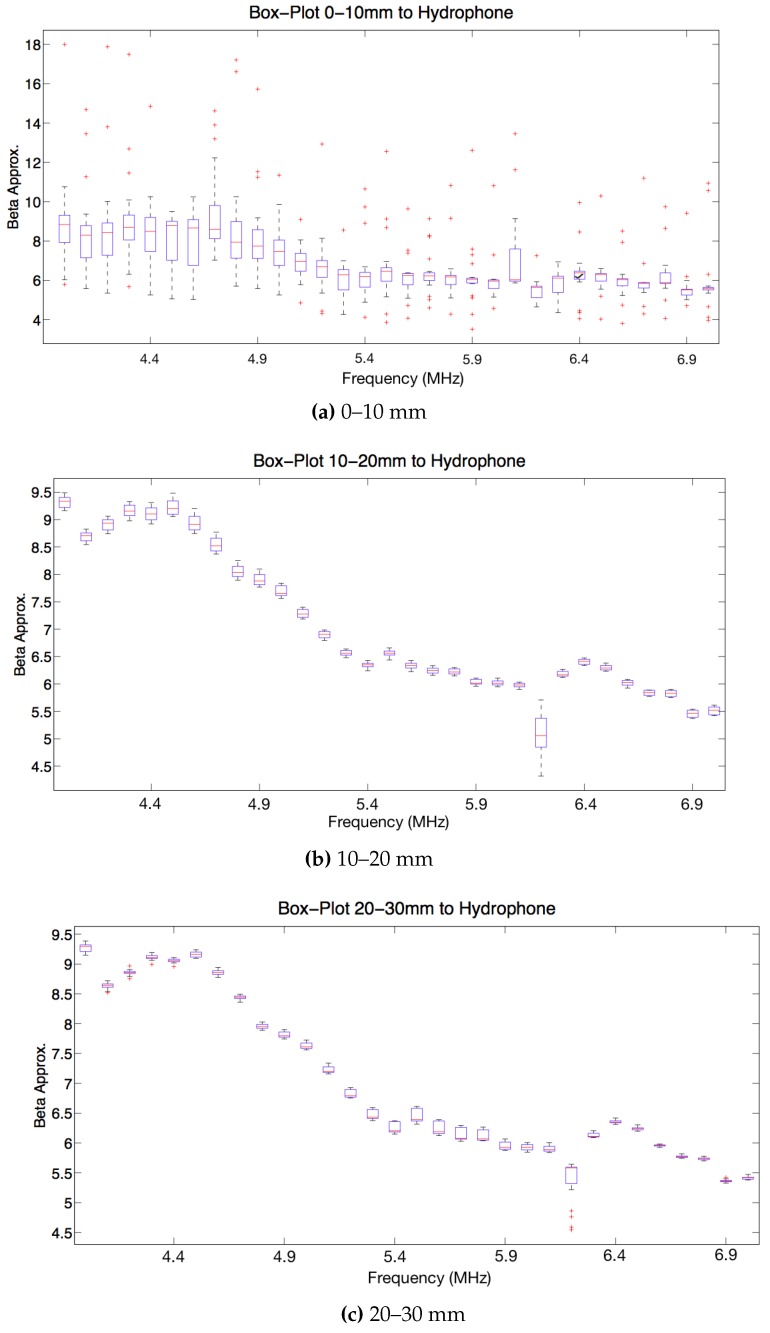
Box-plot beta versus frequencies from 4 MHz to 7 MHz in steps of 0.1 MHz and using 50 cycles. Mean and standard deviation values are computed considering different distances specimen-hydrophone: in (**a**) distance 0–10 mm, in (**b**) distance 10–20 mm and in (**c**) distance 20–30 mm.

In the Figure 10a it can be observed that there are a lot of dispersion (it can be appreciated by the red crosses, which are so far from the mean). A lower level of dispersion in the values of the nonlinear parameter β is shown in the Figure 10b. The next Figure 10c reveals a similar level of dispersion to the previous figure. Attending to the center frequency of the transducer, there are a range in which the beta mean is stable and the variance is low. It is between 5.7 MHz and 6.1 MHz and it was selected 5.9 MHz because it is a central value of this range and it is far from 6.2 MHz. In this last frequency the value of beta is different from the previous and posterior frequency.

#### 3.1.3. Cycles and Distance

**Figure 11:**
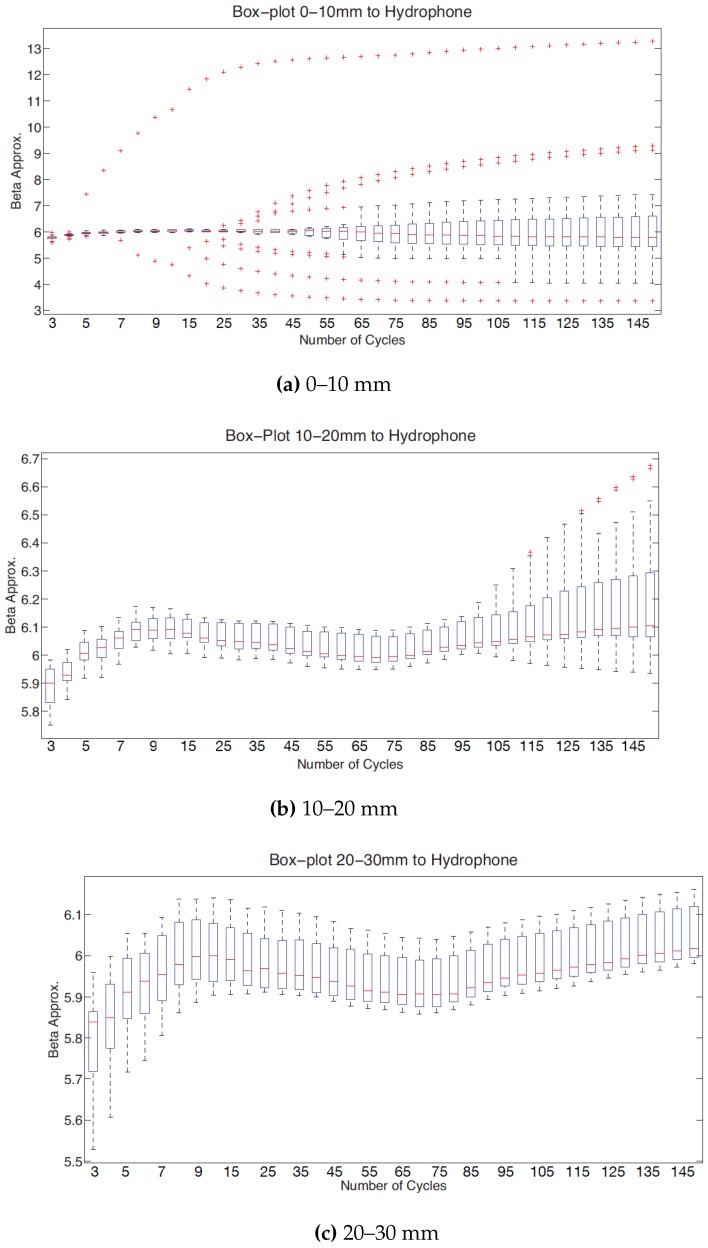
Box-plot beta versus cycles using a frequency of 5.9 MHz. Mean and standard deviation values are computed considering different distances specimen-hydrophone: in (**a**) distance 0–10 mm, in (**b**) distance 10–20 mm and in (**c**) distance 20–30 mm.

With the excitation level and frequency fixed, it will be selected the distance between specimen and hydrophone and the number of cycles analysed in which the measurement is suitable. In Figure 10a and Figure 11a, with a distance between 0–10 mm from the specimen to the hydrophone, there is too much dispersion because of the interferences caused by the small layer of water between specimen and hydrophone. These figures do not provide important information.

The two next Figure 11b,c have a similar pattern, the value of the β parameter increases in the first section, then decreases, then remains stable for a stretch and finally increases. The only difference between them is that there are interferences observed with less distance (10–20 mm). This interference affects from 100 cycles analysed. This provides that this material is very homogeneous because it can be analysed a lot of cycles without interferences.

There is a wide range in which the mean of non-linear parameter is stable (between 50–80 cycles). It is reasonable to choose this range because there are not interferences and the mean of the nonlinear parameter β is approximately constant. By increasing the number of cycles, the received signal contains information of the internal bounces in the specimen, which influences the β parameter.

With the same box-plot it can be deduced the appropriate distance to do a feasible measurement. This could be fitted in the range of 10–30 mm because in this range, for the number of cycles fixed previously, there are not interferences and the value of beta is stable. Within this range, for a shorter distance the attenuation is lower than in a longer distance. Therefore it is desirable to choose a distance close to 10 mm.

#### 3.1.4. Selected Parameters

The parameters chosen for PMMA material are shown in Table 1 for the configuration established in this paper.

### 3.2. CFRP Results

#### 3.2.1. Excitation Level

As with PMMA material, the excitation level was chosen in a first approximation with three frequencies (4 MHz, 5 MHz and 6 MHz), which are different from PMMA frequencies, around the center frequency of the transducer and three different energies (320 mV, 240 mV and 160 mV). A scan was performed in the wave emission direction, between 0.5 mm and 50.5 mm from the specimen.

**Figure 12:**
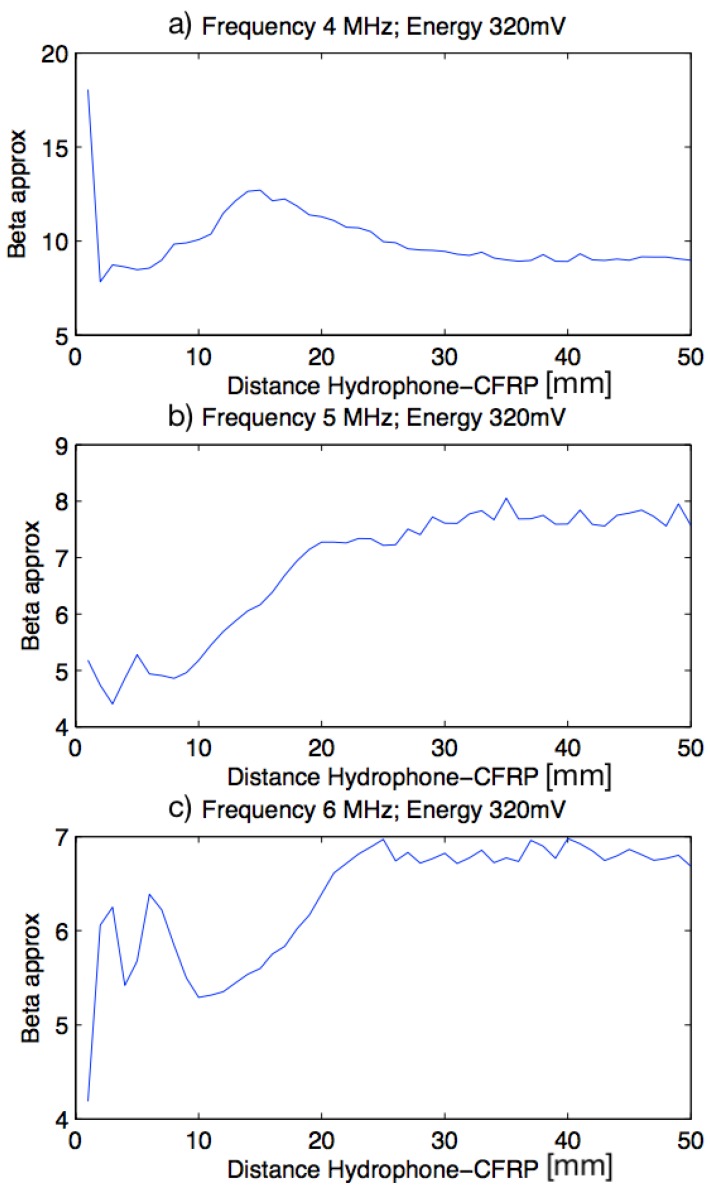
Plots beta versus distance specimen-hydrophone. (**a**) Frequency of 4 MHz and Energy of 320 mV; (**b**) Frequency of 5 MHz and Energy of 320 mV; (**c**) Frequency of 6 MHz and Energy of 320 mV.

In the Figure 12 it can be observed that the nonlinear parameter β in the first two and a half centimetres has a very high variation. From there, variation is established around a value of beta and this stays until the end of the scanning. Results show the same pattern with a fixed frequency and for the different values of excitation level. By this reason, the excitation level of 320 mV was chosen by the same reason given with PMMA material.

#### 3.2.2. Frequency

After choosing the excitation level, it was studied data from the tests with box-plots. It was analysed the value of the non-linear parameter β in different intervals of distance specimen-hydrophone: between 15–20 mm, 20–25 mm and 25–30 mm, for 4 and 20 cycles analysed and for the different frequencies (4–7 MHz) to get information about which frequency has less variance around the central frequency of the transducer.

**Figure 13:**
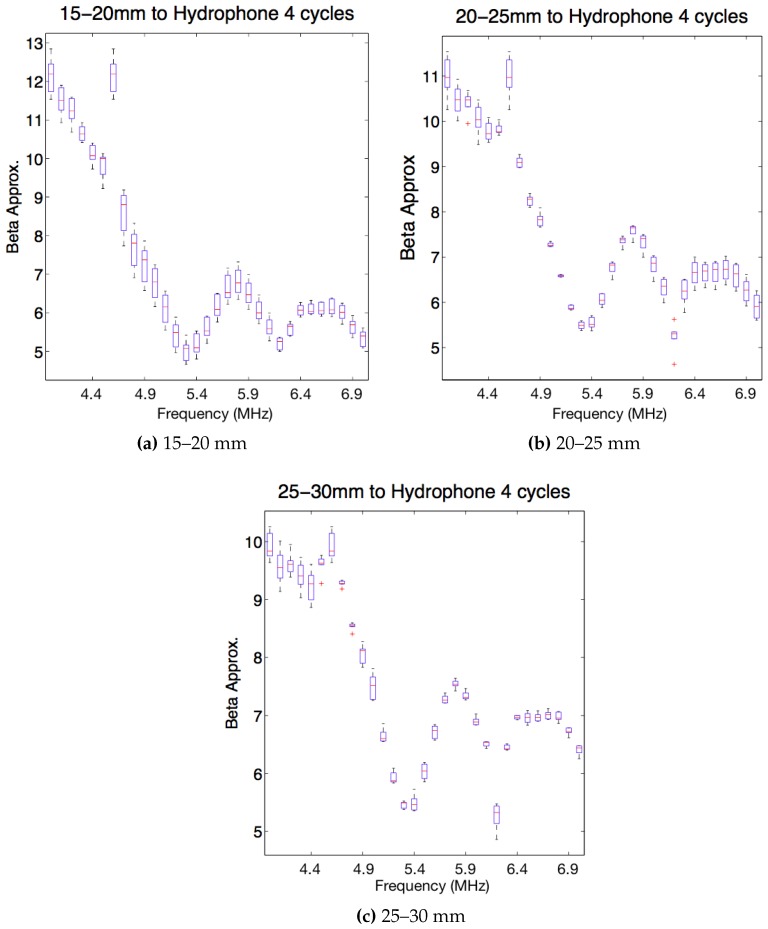
Box-plot beta versus frequencies from 4 MHz to 7 MHz in steps of 0.1 MHz for 4 cycles. Mean and standard deviation values are computed considering different distances specimen-hydrophone: in (**a**) distance 15–20 mm, in (**b**) distance 20–25 mm and in (**c**) distance 25–30 mm.

In the Figure 13a it can be observed that there are a lot of dispersion with 4 cycles. In the next Figure 13b, there is also dispersion although less than in the distance of 15–20 mm. On the other hand, in the third image Figure 13c, the dispersion is lower than in the previous two images. With 4 cycles the values of β with the frequency follow the same pattern for the three different distances. For 4 cycles, the value of the frequency for which the value of beta converges after several tests is 5.8 MHz.

#### 3.2.3. Cycles and Distance

**Figure 14:**
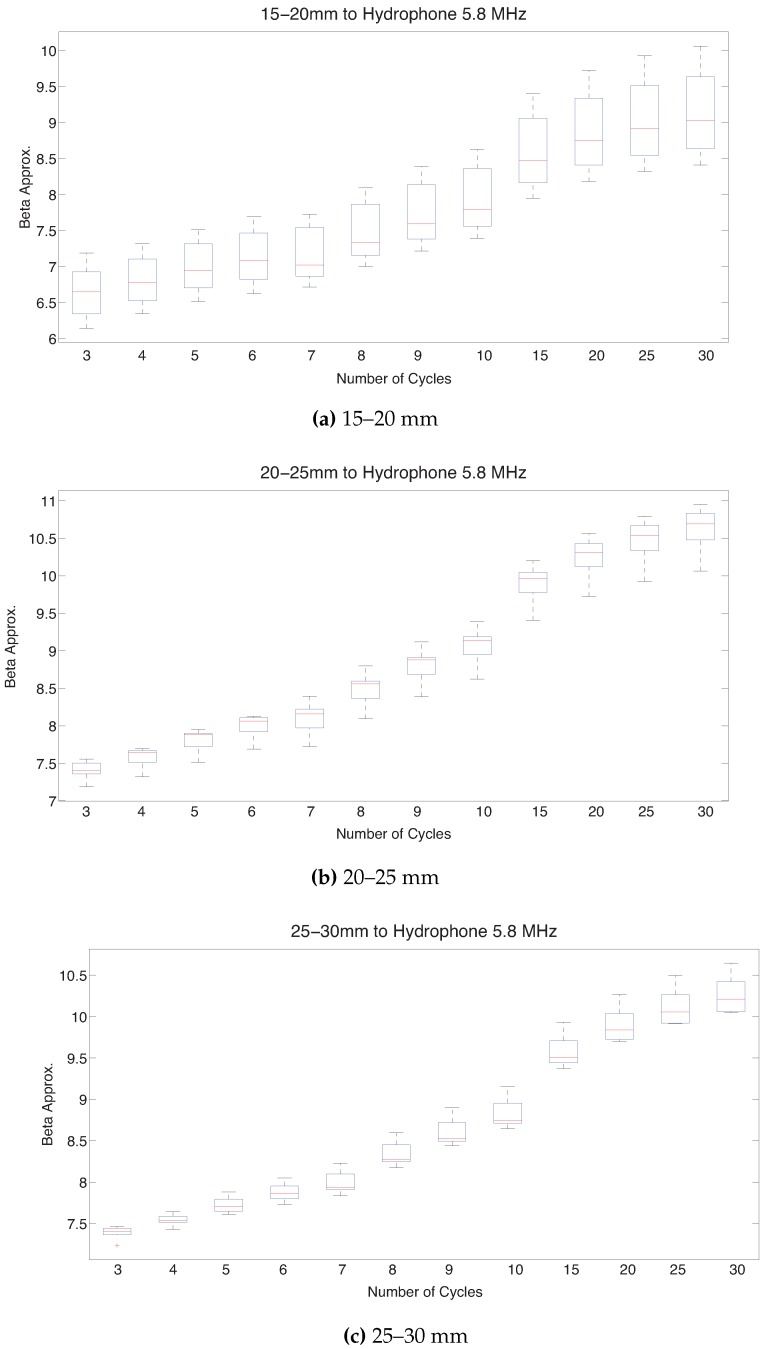
Box-plot beta versus cycles using a frequency of 5.8 MHz. Mean and standard deviation values are computed considering different distances specimen-hydrophone: in (**a**) distance 15–20 mm, in (**b**) distance 20–25 mm and in (**c**) distance 25–30 mm.

With the fixed excitation level and frequency, in the same way as PMMA, it can be selected the distance between specimen and hydrophone and the number of cycles analysed in which the measurement is suitable. In the next three figures it can see, for different distances (15–20, 20–25 and 25–30 mm from specimen) mean and percentiles of beta versus several number of cycles analysed (3–30 cycles), Figure 14.

Dispersion values decreases with increasing distance to the specimen and increase with the number of cycles due to the interferences specimen-hydrophone. It was selected 4 cycles because with these cycles there is not interferences and the interferences are lower than other number of cycles.

With the last box-plot it can be deduced the appropriate distance to do a feasible measurement. This could be fitted in the range of 25–30 mm because in this range, for the number of cycles fixed previously (4 cycles), there are not interferences and the value of beta has lower dispersion than with other distances. Within this range, for a shorter distance the attenuation is lower than in a longer distance. Therefore it is desirable to choose a distance near to 25 mm.

#### 3.2.4. Selected Parameters

The parameters chosen for CFRP material are shown in Table 2 for the configuration established in this paper.

Parameters set in the table are advisory and more test have to be done as it is an anisotropic material. Because of the frequencies used, bounces occur in the layers because the wavelength is similar to the thickness of each layer.

### 3.3. Application of the Semi-Analytical Approach with PMMA Specimen

An application of the semi-analytical approach has been developed in this section in order to validate the method, comparing the results obtained in this method with the results found in literature.

**Figure 15:**
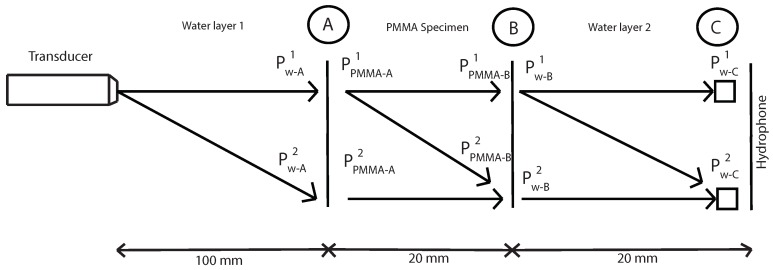
Semi-analytical approach used to extract the nonlinear material’s properties from the measurements in PMMA specimen.

After determining the correct parametric configuration to measure the nonlinear parameter β in an immersion tank with PMMA specimen, measurements using the defined parameters have been carried out. The scheme used for determining the nonlinear parameter β in PMMA is shown in Figure 15.

The distance between transducer and PMMA specimen is 100 mm in order to avoid the near field. The near field distance (NF) is determinated as follows:

$$NF=\frac{a^{2}}{\lambda }$$

where *a* = 5 mm is the radius of the transmitter and λ is the sound wavelength.

$$NF=\frac{a^{2}}{\lambda }=\frac{{\left(5\;\mathrm{mm}\right)}^{2}}{\frac{1500\cdot 10^{3}\;\mathrm{mm}/s}{5.8\cdot 10^{6}\;\mathrm{Hz}}}=96.6\;\mathrm{mm}$$

The distance between specimen and hydrophone has been chosen considering the results obtained in Table 1.

The values taken to measure the nonlinear parameter β in the immersion tank are: excitation level 320 mV, frequency 5.8 MHz, distance specimen-hydrophone 20 mm and 50 cycles were analysed.

The first step is to determinate the nonlinear parameter β and geometric attenuation between B and C points with fundamental and second harmonics pressures in this points without PMMA specimen.

$$P_{w-B}^{1}=8.7868\times 10^{-9}\;m\;P_{w-B}^{2}=1.0985\times 10^{-9}\;m\;P_{w-C}^{1}=7.5771\times 10^{-9}\;m\;P_{w-C}^{2}=1.0131\times 10^{-9}\;m$$

**Figure 16:**
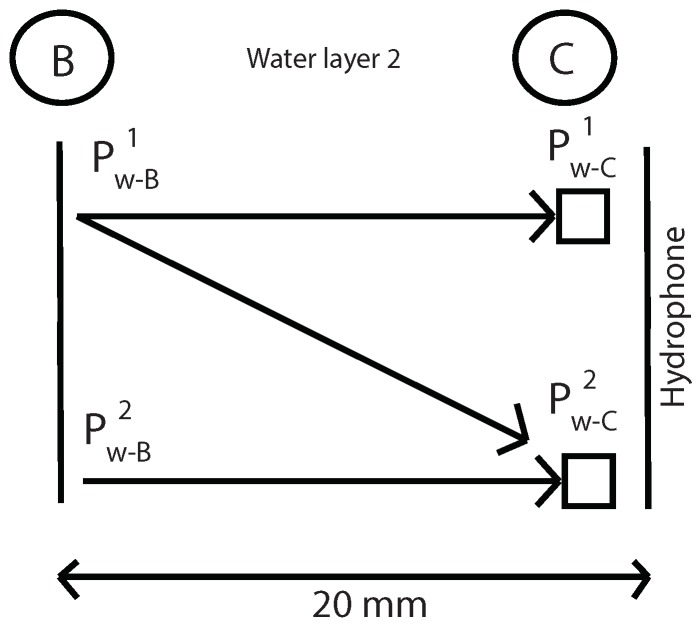
Determination of the β parameter and geometric attenuation in water layer 2 without specimen.

With this values and using the Equations (12) and (15), it will be determinated nonlinear parameter β in water and geometric attenuation (Figure 16). Water attenuation value was taken from literature ($\alpha =20\times 10^{-15}\cdot f^{2}$) [32].

The values of beta and geometric attenuation are:

$$\beta_{w}=2.5929\;\alpha_{g}^{BC}=6.7331\frac{\mathrm{dB}}{m\cdot \mathrm{MHz}}$$

The nonlinear parameter β obtained in water is ($\beta_{w}=2.59$). This value differs one unit with the value found in the literature ($\beta_{w}=3.5$) [21].

**Figure 17:**
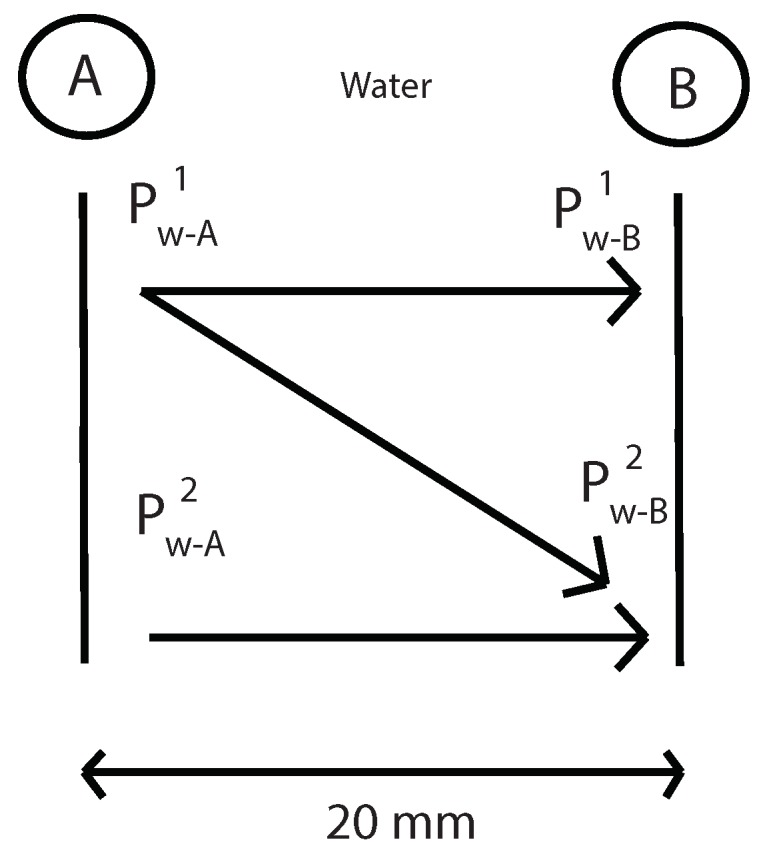
Determination of the geometric attenuation between A and B points without specimen.

Then, using the same Equations (12) and (15), geometric attenuation between A and B point is obtained without the presence of the specimen (see Figure 17). Now, “x” is the distance between A and B.

The values of pressure in A and B points are:

$$P_{w-A}^{1}=1.0255\times 10^{-8}\;m\;P_{w-A}^{2}=1.1695\times 10^{-9}\;m\;P_{w-B}^{1}=8.7868\times 10^{-9}\;m\;P_{w-B}^{2}=1.0985\times 10^{-9}\;m$$

The value of geometric attenuation between A and B points is:

$$\alpha_{g}^{AB}=7.0531\frac{\mathrm{dB}}{m\cdot \mathrm{MHz}}$$

The next step is to measure the fundamental and second harmonic pressure in C with the presence of the PMMA specimen. This values of pressure are:

$$P_{w-C}^{1}=1.9585\times 10^{-9}\;m\;P_{w-C}^{2}=9.9917\times 10^{-11}\;m$$

**Figure 18:**
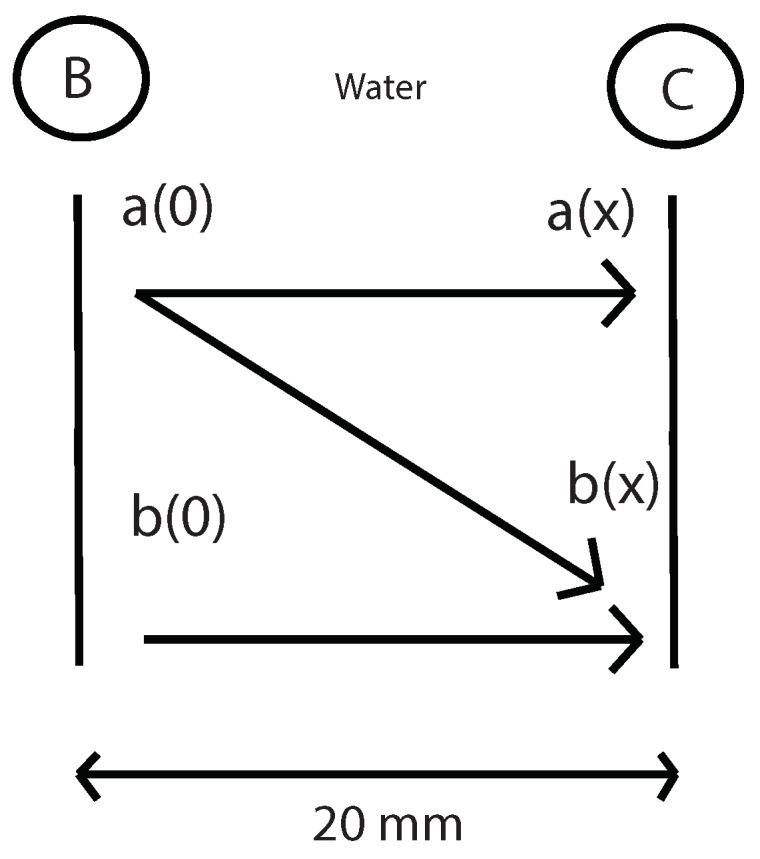
Determination of the fundamental and second harmonic in B by propagating this values from C to B.

With this values and the values of nonlinear parameter β in water and geometric attenuation between B and C (previously calculated), it will be determinated the amplitude of the fundamental and second harmonic in water and in B point (Figure 18), using the equations below:

(24) $$\begin{array}{c} a\left(0\right)=\frac{a(x)}{e^{-(\alpha_{g}+\alpha )x}}=2.2711\times 10^{-9}\;m \end{array}$$

(25) $$\begin{array}{c} b\left(0\right)=\frac{b\left(x\right)-\frac{\beta k^{2}a{\left(0\right)}^{2}}{4(\alpha_{g}-2\alpha )}e^{-(2\alpha +2\alpha_{g})x}}{e^{-(4\alpha +\alpha_{g})x}}=1.2897\times 10^{-10}\;m \end{array}$$

The last step, before reaching the goal, is to obtain the values of the fundamental and second harmonic in A and B in specimen by multiplying this values in water for transmission coefficient water-specimen.

The transmission coefficient water-specimen is:

(26) $$\begin{array}{c} T_{w-s}=\frac{2Z_{w}}{Z_{w}+Z_{s}}=0.6365 \end{array}$$

where $Z_{w}$ and $Z_{s}$ are the water impedance and the specimen impedance respectively.

Finally, with the values of fundamental and second harmonic in A and B in specimen and taking specimen attenuation from literature and geometric attenuation calculated before between A and B points, the nonlinear parameter β in specimen, considering specimen and geometric attenuation, is calculated as follows:

$$P_{PMMA-A}^{1}=6.5277\times 10^{-9}\;m\;P_{PMMA-A}^{2}=7.4444\times 10^{-10}\;m\;P_{PMMA-B}^{1}=1.6657\times 10^{-9}\;m\;P_{PMMA-B}^{2}=9.4592\times 10^{-11}\;m$$

(27) $$\begin{array}{c} \beta =\frac{\left(b\left(x\right)-b\left(0\right)e^{-(4\alpha_{s}+\alpha_{g})x}\right)\left(4\left(\alpha_{g}-2\alpha_{s}\right)\right)}{k^{2}a{\left(0\right)}^{2}e^{-(2\alpha_{s}+2\alpha_{g})x}}=7.84 \end{array}$$

where “x = 20 mm” is the specimen’s thickness and $\alpha_{s}=4.64\cdot \frac{\mathrm{dB}}{m}$ is the PMMA attenuation obtained using the Equation (23).

The nonlinear parameter β in PMMA obtained, removing water contribution and considering specimen and geometric attenuation is similar to the values found in literature ($\beta_{PMMA}=7.5$) [30].

## 4. Conclusions

Parameters to measure nonlinearity (nonlinear parameter β) in PMMA and CFRP materials have been determined by tests in an immersion tank with water as a medium. The nonlinear parameter β has been determined using the variation of the finite amplitude method with harmonic generation. Using this as a reference, it has been deducted the experimental configuration necessary to measure this nonlinear parameter in a correct and feasible way.

For the PMMA material the experimental configuration was deducted. The excitation level of 320 mV was chosen because the higher excitation level, the higher nonlinearity level obtained (β parameter) and consequently greater sensitivity to detect material damage. Harmonics of greater amplitude are obtained with a higher level of energy. The separation between the transducer and the specimen was established as 100 mm in order to avoid the near field of the transducer. The distance between the specimen and the hydrophone was determined in a range of 10–30 mm. The first centimeter is eliminated since rebounds, produced in the water layer between the specimen and the hydrophone, produce constructive and destructive interference in the signal and this results in falsified values of the β parameter. It was found that the correct number of cycles to get a correct value of the nonlinear parameter was between 50–80, due to the mean of the nonlinear parameter β is approximately constant in that stretch. By increasing the number of cycles, the received signal contains information of the internal bounces in the specimen, leading in β values with noise. Finally, the frequency was fixed at 5.9 MHz, close to the resonance frequency of the transducer. The selected frequency is within the range (5.7 to 6.1 MHz) in which the β mean is stable and the variance is low.

On the other hand, in carbon fibre reinforced polymer (CFRP) plate the values of the determined experimental configuration parameters are as follows. The distance between the transducer and the specimen was established at 100 mm, the same as PMMA. This is caused by the influence of the near field that it is wanted to avoid. The separation between the specimen and the hydrophone was determined at a range of 25 mm to 30 mm, choosing the nearest values due to the same reason as well as in PMMA material. The number of cycles in this case descends to 4 cycles, because of the interferences produced inside the material. The thickness of the fiber layers, two orders of magnitude less than the thickness of the PMMA, causes interference in the received signal with fewer cycles. And finally, the frequency chosen to measure this material is 5.8 MHz, close to the resonance frequency of the transducer and with the smallest standard deviation. Due to the material nature, which is very inhomogeneous, anisotropic and random, the configuration parameters in this material must be determined stronger than in this work.

After obtaining the correct configuration to measure the nonlinear parameter β in PMMA and CFRP in an immersion tank, a semi-analytical approach has been developed in order to determine the nonlinear parameter in any material by removing water contribution and considering viscous and geometric attenuation.

Finally, an application of the semi-analytical approach with PMMA material has been developed in order to validate the method. The result of the nonlinear parameter β in PMMA with this method is 7.84 and this parameter appears consistent with the value found in literature, β=7.5 [30].

## Figures and Tables

**Table 1:** Selected parameters to measure the nonlinear parameter in PMMA.

Material	Excitation Level (mV)	Frequency (MHz)	Cycles	Distance (mm)
PMMA	320	5.9	50–80	10–30

**Table 2:** Selected parameters to measure the nonlinear parameter in CFRP.

Material	Excitation Level (mV)	Frequency (MHz)	Cycles	Distance (mm)
CFRP	320	5.8	4	25–30

## Abbreviations

The following abbreviations are used in this manuscript:

PMMA	Polymethylmethacrylate
CFRP	Carbon Fiber Reinforced Polymer
FAM	Finite Amplitude Method
NEWS	Nonlinear Elastic Wave Spectroscopy
NRUS	Nonlinear Resonant Ultrasound Spectroscopy
NWMS	Nonlinear Wave Modulation Spectroscopy
DAE	Dynamic Acousto-Elasticity
NDT	Nondestructive Testing
SAW	Surface Acoustic Waves
